# Kilohertz frequency nerve block enhances anti-inflammatory effects of vagus nerve stimulation

**DOI:** 10.1038/srep39810

**Published:** 2017-01-05

**Authors:** Yogi A. Patel, Tarun Saxena, Ravi V. Bellamkonda, Robert J. Butera

**Affiliations:** 1 Georgia Institute of Technology, Department of Biomedical Engineering, Atlanta, GA, 30332 USA; 2 Georgia Institute of Technology, Bioengineering Graduate Program, Atlanta, GA, 30332 USA; 3 Georgia Institute of Technology, Neural Engineering Center, Atlanta, GA, 30332 USA; 4 Duke University, Department of Biomedical Engineering, Durham, NC 27708 USA; 5 Georgia Institute of Technology, School of Electrical and Computer Engineering, Atlanta, GA 30332 USA

## Abstract

Efferent activation of the cervical vagus nerve (cVN) dampens systemic inflammatory processes, potentially modulating a wide-range of inflammatory pathological conditions. In contrast, afferent cVN activation amplifies systemic inflammatory processes, leading to activation of the hypothalamic-pituitary-adrenal (HPA) axis, the sympathetic nervous system through the greater splanchnic nerve (GSN), and elevation of pro-inflammatory cytokines. Ideally, to clinically implement anti-inflammatory therapy via cervical vagus nerve stimulation (cVNS) one should selectively activate the efferent pathway. Unfortunately, current implementations, in animal and clinical investigations, activate both afferent and efferent pathways. We paired cVNS with kilohertz electrical stimulation (KES) nerve block to preferentially activate efferent pathways while blocking afferent pathways. Selective efferent cVNS enhanced the anti-inflammatory effects of cVNS. Our results demonstrate that: (i) afferent, but not efferent, cVNS synchronously activates the GSN in a dose-dependent manner; (ii) efferent cVNS enabled by complete afferent KES nerve block enhances the anti-inflammatory benefits of cVNS; and (iii) incomplete afferent KES nerve block exacerbates systemic inflammation. Overall, these data demonstrate the utility of paired efferent cVNS and afferent KES nerve block for achieving selective efferent cVNS, specifically as it relates to neuromodulation of systemic inflammation.

Activation, inhibition, and control of the innate immune system is vital for maintenance of homeostasis in living organisms, and one in which both the central (CNS) and peripheral nervous systems (PNS) play a critical role. The CNS actively responds to acute immune challenges by altering body temperature, stimulating the HPA axis, as well as up- and down-regulating specific sympathetic pathways, which are primarily involved in attenuating both cellular and humoral responses initiated by an immune challenge. The PNS enables modulation of the response to an immune challenge by allowing directional stimulation of nerves involved in signaling between the CNS and effector peripheral targets (ganglia, organs, tissues). Specifically, studies from over the last two decades have highlighted the ability to modulate the systemic response to an immune challenge, both in animal and clinical investigations, by electrical stimulation of the cVN.

Studies first conducted by Borovikova *et al*.^1^ demonstrated down regulation of the systemic response to lethal endotoxemia in rats by electrically stimulating the efferent pathways in the cVN (achieved by stimulating the distal end of the transected cVN). Their results demonstrated the direct influence of the response to an incoming and ongoing acute immune challenge via electrical stimulation. These initial findings have led to a significant number of investigations aimed at the use of cVNS for modulation of inflammation in a variety of clinical conditions (ClinicalTrials.gov Identifiers: NCT01552941, NCT02311660). Continued investigations^2^ into the mechanism of action have engendered the cholinergic anti-inflammatory pathway. We refer the reader to Martelli *et al*.^3^ for a critical review of the cholinergic anti-inflammatory pathway.

Although the mechanism is unknown and requires significant inquiry, results from both animal and initial clinical investigations posit a potential benefit of efferent cVNS in ameliorating systemic and local inflammation. Many, if not all, cVNS investigations stimulate the intact cVN, leading to activation of both afferent and efferent pathways, or achieve stimulation of afferent or efferent pathways by transecting the nerve. While these approaches are sufficient for elucidating acute effects in animal investigations, clinical translation of selective afferent (acVNS) or efferent cVNS (ecVNS) requires a safe and effective alternative approach. Various studies have investigated the ability to selectively stimulate via custom electrode geometries and different stimulation waveforms. These approaches suffer from clinical challenges such as patient-to-patient variations in nerve anatomy as well as surgical placement and movement of electrodes. Furthermore, a cervical vagotomy is not desirable in clinical settings due to the fact that a significant amount of parasympathetic control is exerted on the viscero-motor systems through the vagi^4^,^5^,^6^. A safe, effective, and reversible selective acVNS or ecVNS method is clearly necessary for controlling inflammation in humans.

We have previously shown that sinusoidal kilohertz electrical stimulation (KES) enables a safe, robust, and rapidly reversible block of nerve activity in the cVN^7^. We thus hypothesized that KES nerve block could be utilized to achieve a quick, reliable, and temporary virtual vagotomy for inhibiting activation of afferent pathways while delivering ecVNS. A KES-enabled virtual vagotomy has many advantages over uncontrollable and irreversible procedures such as nerve transection or pharmacological blockade presently used in both scientific and clinical applications. The investigation presented here demonstrates a paradigm for selective ecVNS and afferent KES nerve block for suppression of systemic inflammation in response to bacterial lipopolysaccharide (LPS)-induced endotoxemia in a rat animal model (Fig. 1A–C). We quantified both nerve activation and inhibition through electrophysiological recordings of peripheral nerve activity along with biochemical changes induced by cVNS and KES nerve block.

Our results demonstrate that when the virtual vagotomy is successfully employed, the anti-inflammatory benefits of ecVNS are enhanced. In contrast, when the virtual vagotomy is incomplete, the beneficial effects of ecVNS are partial. Collectively, this acute study demonstrates (i) the ability of KES nerve block to provide a method for virtually transecting nerves safely, robustly, and reversibly; (ii) paired delivery of ecVNS and afferent KES nerve block for modulation of systemic inflammatory processes; and (iii) quantitative criteria for evaluating the status of KES nerve block. This technique of paired delivery for achieving selective acVNS or ecVNS may benefit on-going investigations utilizing cVNS, specifically when developing human bioelectronic medicines based upon laboratory findings.

## Results

### Afferent cVNS synchronously activates the greater splanchnic nerve

**Figure 1:**
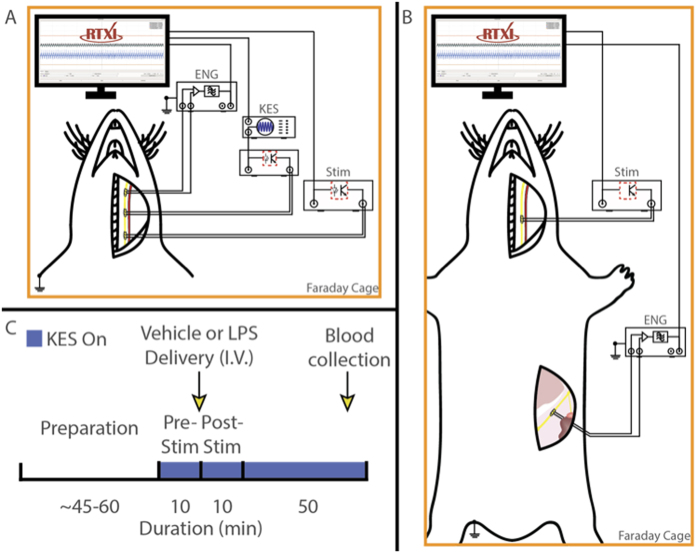
(**A**) Experiment setup and electrophysiological configuration. The left cVN was exposed and fitted with three cuff electrodes. ENG measurements were made from the cranial end of the exposed nerve. cVNS was delivered to the caudal end of the exposed nerve, with a KES delivering electrode located cranially. (**B**) Preparation used for to measure ENG from the GSN. (**C**) Experiment timeline. Nerve and electrode preparation were followed by a 10 minute stimulation (pre-stim) period, in which either cVNS or cVNS + KES were delivered to the nerve. Vehicle or LPS was injected through the lateral tail vein, followed by another 10 minute stimulation (post-stim) period. For nerve block experiments, KES was on for the entire 70 minutes. Blood was collected 50 minutes after the post-stim period for biochemical analysis.

**Figure 2:**
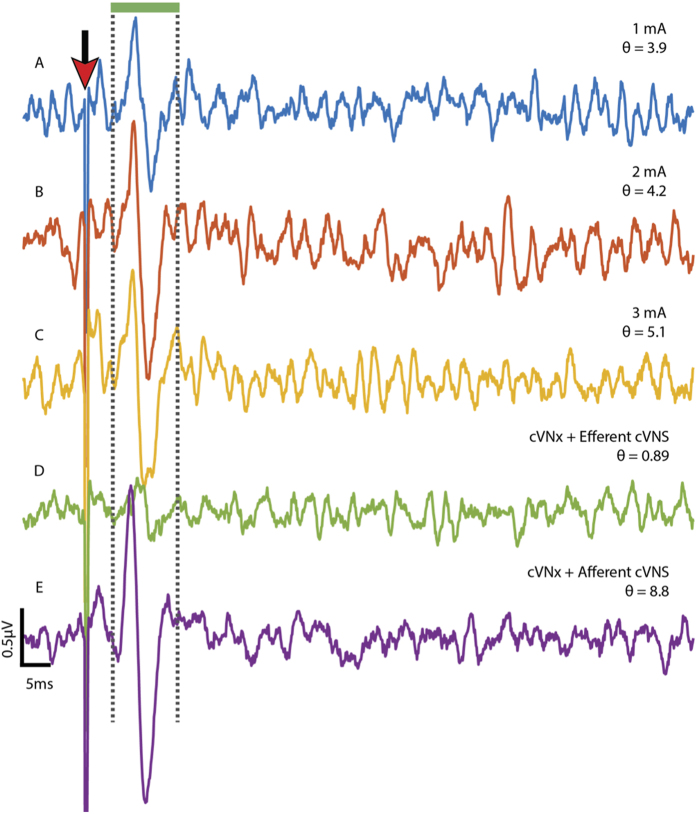
cVNS activates the GSN in a synchronous and dose-dependent manner. (**A**–**C**) Increasing stimulus intensities are delivered to the intact left cVN. Simultaneous ENG measurements are made on the ipsilateral GSN. (**D**) The caudal end of the transected cVN is stimulated, activating efferent pathways alone. (**E**) A cVNx is performed and the cranial end of the cVN is stimulated, activating afferent pathways. Waveforms shown are stimulus-triggered averages from 1000 stimulation trials. The red arrow indicates stimulus artifact, and *θ* values are presented for each waveform.

Afferent activation of the cVN has been shown to up-regulate pro-inflammatory signaling via Interleukin-1 beta (IL-1*β*) expression and activation of the HPA axis through the GSN^8^,^9^. Prior to conducting KES nerve block experiments, we conducted a small set of experiments (n = 3 rats) to assess GSN activation as a function of cVNS as well as the effects of acVNS on inflammatory tumor necrosis factor alpha (TNF-*α*) expression. cVNS was delivered to the left cVN and electroneurogram (ENG) measurements were made from the GSN with biphasic stimulation intensities of 1, 2, and 3 mA_pp_ (1 Hz, 0.4 ms) (Fig. 1B). A cVNx was performed afterwards on either the cranial or caudal end of the electrode for acVNS or ecVNS. A total of 1000 stimuli were delivered at each amplitude in each configuration to enable detection of the evoked GSN activity. Increasing amplitude evoked GSN potentials with latencies of 5–8 ms were measured during cVNS (Fig. 2A–C). A linear fit of the *θ* calculations revealed a direct relationship between stimulation intensity and evoked GSN activity (R^2^ = 0.94). Stimulus-triggered averages from either ecVNS (Fig. 2D) or acVNS (Fig. 2E), along with *θ* calculations, demonstrated that cVNS-induced activation of the GSN occurs during acVNS only. Biochemical analysis to quantify TNF-*α* expression (data not shown) demonstrated an increase in serum TNF-*α* even without LPS delivery.

### Paired efferent cVNS and complete afferent KES nerve block enhance anti-inflammatory effects

**Figure 3:**
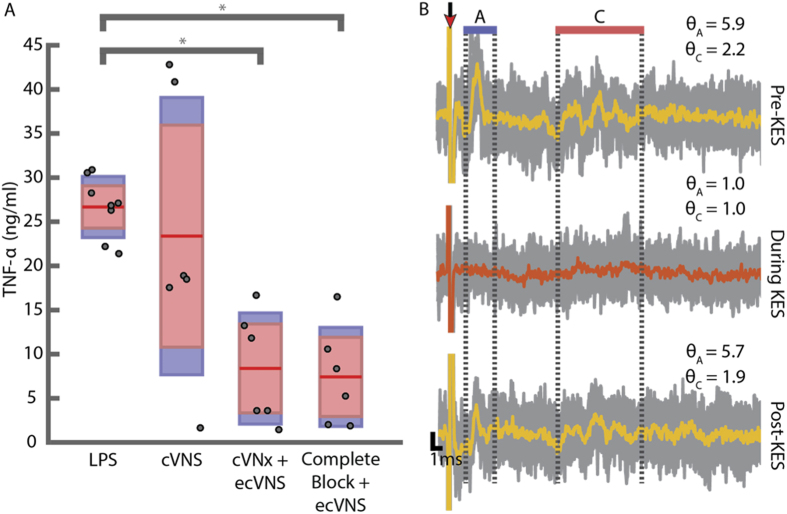
TNF-*α* expression and ENG data for baseline cVNS conditions. (**A**) TNF-*α* levels from animals receiving no stimulation (LPS, n = 8), stimulation of the intact cVN (cVNS, n = 6), vagotomized efferent cVNS (cVNx + ecVNS, n = 6), and complete afferent KES nerve block with paired efferent cVNS (Complete Block + ecVNS, n = 6). Asterisks denote significance between bracketed groups (*α* = 0.05). (**B**) Representative recordings from the caudal end of the cVN pre-, during-, and post-KES delivery. Average measurements (yellow and orange) are superimposed upon individual runs (grey). (**A** and **C**) Component regions depict the windows used for quantifying nerve activation (*θ*) and block efficacy (*θ*) for (**A** and **C**) components in the ENG measurements. Post-KES averages are from 10 runs only. Red arrow indicates stimulus artifact.

cVNS of the intact cVN leads to bidirectional activation of the vagus, as shown through ENG measurements from locations both cranial and caudal to the stimulation electrode (Fig. 3B,C). Cranial measurements depict two distinct components of the compound action potential (CAP) representing the set of A and C fibers respectively (Fig. 3B). Characterization of *θ* for each component demonstrates substantial activation of both afferent and efferent pathways. cVNS alone did not demonstrate anti-inflammatory effects in all animals receiving LPS injections. No significant difference was determined between control animals (LPS only) and animals receiving LPS injections with cVNS. In contrast, as previously reported by others^1^, cVNx + ecVNS resulted in a statistically significant decrease in TNF-*α* expression (Fig. 3A).

**Figure 4:**
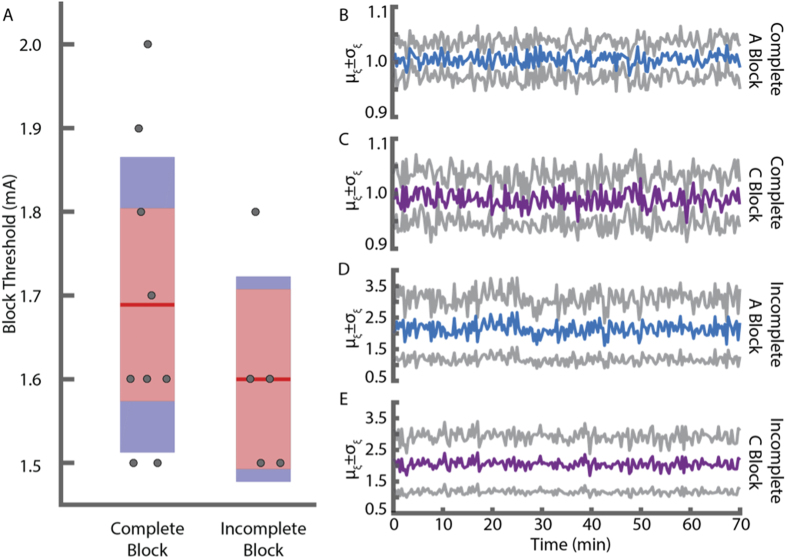
KES nerve block thresholds and *θ* computations. (**A**) KES block thresholds were determined during experimental preparation. Post-hoc analysis of ENG measurements and quantification of *θ* led to sorting of block thresholds into complete and incomplete block groups. Experiments with trials containing *θ* greater than the RMS noise floor during KES delivery were categorized as incomplete block for both biochemical and electrophysiological analysis. (**B**–**E**) Mean and standard deviation of *θ* throughout experiments with both complete afferent KES nerve block ((**B**,**C**) n = 9 from all experiments) and incomplete afferent KES nerve block ((**D**,**E**) n = 5 from all experiments). Recordings from the 70 minute experiments were parsed into 210 trials, each represented by a stimulus-triggered average waveform. Colored traces represent the mean from all experiments in each group, with the grey traces representing ±1*σ*. Experiments with complete afferent KES nerve block met the *θ* criteria, while incomplete block experiments did not.

We utilized KES nerve block with ecVNS of the intact cVN to inhibit activation of afferent pathways while maintaining activation of efferent pathways. ENG measurements from the cranial end of the cVN and biochemical results are shown in Fig. 3. Complete afferent KES nerve block + ecVNS significantly lowered TNF-*α* levels compared to control (LPS only), but not compared to cVNx + ecVNS (Fig. 3A) suggesting the presence of a virtual vagotomy of the cVN with KES. ENG measurements from the cranial end of the cVN were used to calibrate and assess the status of afferent KES nerve block. Sample ENG measurements are shown in Fig. 3B, along with the calculated *θ* values, which indicate complete block of both A and C fiber components. The values at which block was achieved in these experiments, referred to as block thresholds, are depicted in Fig. 4A.

We further investigated *θ* by analyzing its status throughout the course of the experiment. ENG measurements from each experiment were parsed into 210 trials (see Methods). *θ* was calculated for each trial for each experiment, resulting in a time series representation of *θ* with a sampling interval of 20 s. The *θ* mean ± one standard deviation for A and C fiber components across all complete KES nerve block experiments are shown in Fig. 4B,C. For complete KES nerve block experiments, the *θ* criteria for highly efficacious and complete block was met. Post-experiment evaluation of nerve viability demonstrated components as seen in baseline measurements (Fig. 3B).

### Paired efferent cVNS and incomplete afferent KES block lead to pro-inflammatory effects

**Figure 5:**
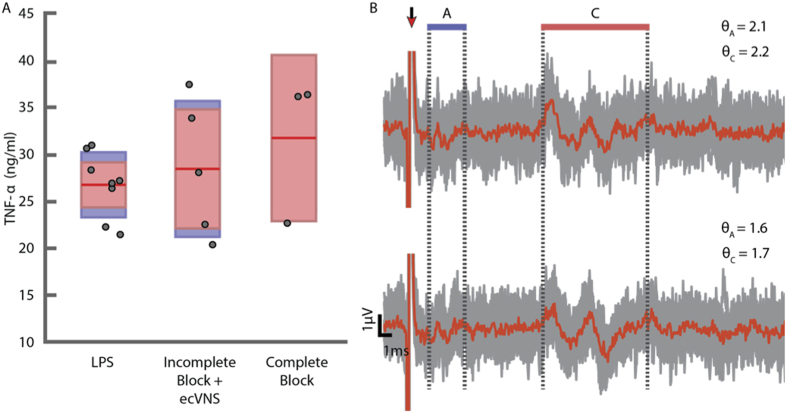
Incomplete afferent KES nerve block and KES nerve block alone are not sufficient for activating anti-inflammatory pathways. (**A**) TNF-*α* expression from control (LPS, n = 8), incomplete afferent KES nerve block and paired ecVNS (Incomplete Block + ecVNS, n = 5), and complete afferent KES nerve block only (Complete Block, n = 3). (**B**) Example ENG measurements from the caudal end of the cVN during incomplete afferent KES nerve block. Average waveforms (red) are superimposed upon individual runs (grey), with *θ* presented for each CAP component. Red arrow indicates stimulus artifact. This example is from an experiment in which the calibration period was successfully completed, however post-hoc analysis revealed that afferent KES nerve block was incomplete. The A fiber component is partially blocked, however not complete, and the C fiber component is unmodified compared to baseline measurements.

Although the initial calibration tests were successful, a subset of experiments (n = 5 rats) were found to be incomplete with respect to afferent KES nerve block. Post hoc analysis revealed that the criteria for complete KES nerve block were not met in these animals, as represented in the sample ENG measurements and calculated *θ* values shown (Fig. 5B). Both A and C components are present in stimulus-triggered average waveforms. Furthermore, TNF-*α* expression was elevated in the incomplete KES nerve block experiments to values similar to control (LPS only, Fig. 5A). The block thresholds used in incomplete KES nerve block experiments are shown in Fig. 4A. To investigate why KES nerve block was sometimes incomplete, we characterized *θ* over the 70 minute experiment period. Time series representations of *θ* for all incomplete KES nerve block experiments were generated. The *θ* mean ± one standard deviation for A and C fiber components across all incomplete KES nerve block experiments are shown in Fig. 4D,E. Compared to complete KES nerve block, *θ* time series for incomplete KES nerve block presented a greater mean and standard deviation. No distinguishing features or events were observed suggesting why KES nerve block failed, however.

### Virtual vagotomy alone does not provide anti-inflammatory benefits of cVNS

Previous reports^1^ demonstrated that animals receiving cVNx and LPS, but not ecVNS, had elevated serum TNF-*α*, similar to control animals (LPS only). For comparison, we characterized serum in animals receiving complete KES nerve block and LPS, but no ecVNS. Complete KES nerve block was verified using the aforementioned procedures and the same experimental protocol (Fig. 1C) was carried out. ELISA results (Fig. 5A) showed elevated serum TNF-*α* levels, similar to those previously reported in cVNx animals^1^.

## Discussion

Suppression of systemic and local inflammation via eCVNS has the potential to be a powerful clinical strategy. When used on the bench top, investigations typically transect and stimulate the peripheral end of the vagus. In this report, we demonstrate the ability to conduct a virtual vagotomy via KES nerve block, which is feasible at the bedside. Our primary results are that (1) KES can block evoked nerve activity that is equivalent to nerve transection and (2) KES nerve block alone is insufficient for activating the vagal anti-inflammatory pathways. These results have important clinical implications, as it allows for unidirectional electrical activation of the vagus nerve without the need to transect the nerve.

Results from previous investigations have demonstrated activation of the GSN during cVNS as well as the role of the GSN in regulating inflammation^9^,^10^. However this report is qualitative and only demonstrates the presence of an event upon supramaximal stimulation^11^. We quantified activation of the GSN during cVNS using stimulation intensities (1–3 mA_pp_ vs 0.5–2.0 mA) that are commonly used in clinical applications of cVNS^12^,^13^. Our stimulation parameters differ from those use clinically with respect to pulse width (0.4 ms vs 0.25 ms) and frequency (1 Hz vs 10–20 Hz). A direct relationship was found between cVNS stimulation intensity and the resulting sympathetic activation. Transection of the nerve at either cranial or caudal ends of the stimulating electrode revealed that this activation is predominantly due to activation of afferent pathways (Fig. 2). These results suggest that increased stimulation intensities result in greater activation of the GSN, which carries sympathetic activity to a majority of visceral organs responsible for maintenance of homeostasis. It has been shown that chronic SNS activity drives local persistent inflammation leading to deleterious side effects like cachexia and increased blood pressure^14^,^15^. Thus, while direct activation of the sympathetic splenic and splanchnic nerves can offset inflammation, chronic activation of the GSN in patients receiving cVNS is not a clinically viable strategy.

To the best of our knowledge, this is the first report applying KES nerve block as a tool for achieving a virtual vagotomy and selective efferent stimulation. Furthermore, we establish a robust method and criterion, called block efficacy, for evaluating the status of KES nerve block. This approach enables quantitative validation and evaluation of the effects of KES nerve block. Quantification of block efficacy throughout the experiment (Fig. 4B–E) enables detection of changes in block efficacy and thresholds during application. No significant differences were observed in our experimental cases. It is possible that changes in block efficacy and thresholds may present themselves on longer timescales than those employed here.

In addition, although not evaluated here, it is possible that long-term delivery of KES may lead to physiological changes in nerve conduction, as well as excitability of the nerve. In the present study, a post-hoc assessment of nerve viability was conducted as a binary test for ensuring continued conduction of nerve activity post-KES delivery. Post-hoc assessment (described in Methods section) was conducted in each experiment but limited in time due to the need for blood collection. In each experiment, post-hoc assessment successfully resulted in ENG measurements not significantly different than baseline ENG measurements (Fig. 3B).

We previously demonstrated the ability to use KES nerve block for selective block of A or C fiber components in mammalian^7^ and amphibian^16^ animal models. The current investigation employed KES nerve block as an all-or-none technique. It is critical to note, however, that the use of selective KES nerve block may be useful in cases where selective block of A or C fiber activity is desired. Moreover, the mammalian cVN consists of fibers from A, B, and C fiber classes^17^. Our experimental setup, limited by exposed nerve length and electrode spacing, allowed investigation of only A and C fiber components at a macro scale. To validate block of all fiber classes and sub-types, along with selective KES nerve block, larger animal models in which a greater exposed nerve length is attainable is necessary.

In a subset of experiments (n = 5), post-hoc analysis revealed that block was incomplete (Fig. 5), resulting in increased serum TNF-*α*. Although calibration was conducted in each experiments to determine block threshold for each experiment (Fig. 4A), and ENG measurements were visualized online, it is possible that failure to maintain block could have occurred from changes at the electrode-tissue interface or stimulation equipment. While it is possible for direct current (DC) to contaminate the effects of KES nerve block, it is unlikely because equipment was calibrated prior to starting KES nerve block. Furthermore, DC contamination leads to damage of nervous tissue and can result in uncontrolled and unwanted amounts of either DC stimulation or DC nerve block^18^.

The experimental methods and data analysis methods used in this study suggest one potential framework for clinical use of KES nerve conduction block. First, application of KES nerve conduction block requires a valid readout with temporal dynamics on the order of milliseconds. In our case, we used ENG measurements from the cVN to directly assess the effects of KES nerve conduction block on evoked cVN activity. Without such a readout, selection of the appropriate KES amplitudes and thresholds is difficult. Second, a baseline of what activity is to be blocked must be set. In the present investigation, we utilized the RMS voltage of evoked CAPs as the activity to block. Finally, the required duration of KES nerve block must be known for each nerve and physiological function of interest. These three elements may be incorporated into an implantable device for chronic use, or could be utilized acutely in patients through on-nerve electrodes with percutaneous leads.

One side-effect previously reported during application of KES nerve block is an initial brief period of asynchronous activation of the nerve^7^,^16^,^19^. This response, coined the onset response, is typically short lived (<100 ms) and occurs immediately after initiating KES nerve block. This asynchronous activation is removed from our recordings by the online filtering and post-hoc stimulus-triggered averaging of ENG measurements. Although not measured in our experiments, it is possible that the onset response was present in the form of laryngeal muscle activation. On-going investigations will attempt to quantify laryngeal activation during KES nerve block of the vagus.

Our studies and results presented here utilized the standard protocol for investigating neuromodulation of systemic inflammation on the left cVN. Additional pilot experiments (unpublished) were carried out to investigate the effects of bilateral cVNS and KES nerve block. These data suggest that no additional benefit could be achieved through bilateral neuromodulation similar to previous reports^20^. We also conducted a pilot study to investigate the necessity of the pre-stimulation period for down-regulation of systemic inflammation. Animals were subject to the same stimulation protocol described above, but without the pre-stimulation period. These additional data suggest that the pre-stimulation period has little to no effect on modulation of LPS-induced systemic inflammation. However, both pilot studies require additional experimentation to confirm significance to these findings.

It is clear that systemic inflammation can be modulated through cVNS, as shown by this report and others. How exactly the nervous system modulates systemic inflammation is a topic currently undergoing significant scientific inquiry. It is valuable to highlight knowns and unknowns about the mechanism of action for modulation of systemic inflammation through cVNS. The cholinergic anti-inflammatory pathway posits that the VN is the efferent arm of the inflammatory reflex. The hypothesized mechanism is that parasympathetic efferent fibers in the VN innervate postganglionic sympathetic splenic neurons in the celiac ganglia with axons in the splenic nerve. Stimulation of efferent cVN pathways leads to modulation of the postganglionic splenic neurons and results in suppression of splenic TNF-*α* production. This mechanism of action has received significant debate due to evidence from anatomical investigations demonstrating little or no direct cholinergic vagal innervation of the spleen^21^, from physiological studies demonstrating the need for intact GSN and splenic nerve^22^, and electrophysiological studies, including this report (Fig. 2), showing no measurable connection between ecVNS, the GSN, or the splenic branch of the GSN^10^.

Alternative hypotheses related to mechanism exist, such as the VN controls splenic nerve activity in an indirect manner through CNS reflex, but not by a direct efferent VN pathway^10^. In addition, we would be remiss to ignore the possibility that the effects of cVNS could be of non-physiological origin, and due to activation of afferent and efferent pathways in synchronous or asynchronous manners that drive physiological function to its limits. These contrasting mechanistic and functional results warrant the need for further investigation into the mechanism of action, especially as cVNS is utilized in clinical settings for long-term treatment of inflammatory conditions.

## Methods

### Animal Preparation

All animal care and procedures were reviewed and approved by the Institutional Animal Care and Use Committee at The Georgia Institute of Technology and all methods were performed in accordance with the relevant guidelines and regulations. *In vivo* experiments were carried out on the left cVN and GSN in adult male Sprague-Dawley rats (Charles River). Animals (311 ± 50 g, n = 65) were anesthetized in a chamber using 5% isoflurane (1 liter/min flow rate). Once recumbent, the animal was maintained at 2–3% isoflurane for 45 minutes, and then at 1.5% isoflurane for the remainder of the experiment. Body temperature was monitored and maintained at 37–40 °C with a rectal temperature probe (TM-3, Warner Instruments, Hamden, CT) and warming pad (COM-11289, SparkFun Electronics, Niwot, CO). Depth of anesthesia was evaluated by pinching the rear footpad. When there was no response, the animal’s neck was shaved and depilated. A midline incision was made and the skin and subcutaneous muscles tissues were retracted via blunt dissection. The salivary glands, sternocleidomastoideus, and omohyoideus were repositioned to allow access to the carotid sheath. The cVN and the common carotid artery were separated using a dissection microscope providing a total exposed cVN length of 1.2–1.4 cm.

For studies requiring access to the GSN, the dorsal surface of the animal was prepared using the same preparation techniques above. An incision was made approximately 1 cm caudal to the 6th false rib and approximately 0.5 cm lateral to the spinous processes. The skin, underlying muscles, and *latissiumus dorsi* were blunt dissected and retracted. The suprarenal gland was identified and blunt dissected apart from the surrounding fat layers. The adrenal nerve was identified and followed proximally to the suprarenal ganglia, which is the proximal end of the greater splanchnic nerve. The greater splanchnic was isolated from surrounding fat and connective tissue. Electrodes (described below) were placed on the cVN for stimulation, block, and recording of nerve activity and, when desired, a recording electrode was placed on the GSN (Fig. 1B). Nerves were not desheathed or dissected. Animals were euthanized at the end of the experiment by thoracotomy done to collect a cardiac blood sample.

### Electrophysiology

All experiments were conducted in a Faraday cage with an electrically floating setup powered by an uninterruptible power supply. A floating ground was established by a 20 G needle inserted into the right gastrocnemius muscle and connected to the table. Control of experimental hardware, delivery of stimuli, and data acquisition were all achieved using The Real-Time eXperiment Interface (RTXI^23^). Custom, bi-polar electrodes were made in-house to stimulate, record, and block activity from the cVN and GSN. In brief, braided stainless steel wires (#793500, A-M Systems, Sequim, WA) were threaded through silicone tubing (#807600, A-M Systems, Sequim, WA), spot-welded to platinum-iridium contact pads and the outer surface of the cuff coated with polydimethylsiloxane (PDMS) for electrical insulation. Electrode impedance (1.2 ± 0.6 kΩ) was characterized at 1 kHz using an impedance conditioning module (FHC Inc., Bowdoin, ME). Both electrical and mechanical characteristics were evaluated prior to electrode reuse. For cVN preparations, the electrode spacing was minimized between the stimulation and block electrodes (0.2 ± 0.1 cm), and maximized between the recording and block electrodes (1.0 ± 0.1 cm).

ENG measurements were differentially measured and amplified with a gain of 10^4^x and filtered with a band-pass of 10^2^–10^4^ Hz (SR560, Stanford Research Systems, Sunnyvale, CA) prior to being digitized at 20 kHz (PCIe-6259, National Instruments, Austin, TX). Biphasic constant current pulses (1 mA_pp_, 0.4 ms, 1 Hz) for nerve stimulation were generated using the RTXI signal generator module and optically-isolated using a linear stimulus isolator (A395, WPI, Sarasota, FL) prior to being delivered to the stimulation electrode. Block of nerve activity was achieved using kilohertz electrical stimuli (KES, ref. 7) generated by a function generator (AFG 3021, Tektronix, Beaverton, OR). The function generator output was optically-isolated using an analog stimulus isolator (Model 2200, A-M Systems, Sequim, WA) prior delivery to the block electrode. KES frequencies and amplitudes were chosen based upon previously demonstrated values for complete block of cVN activity^7^. Timing of KES delivery was controlled by gating the function generator output using RTXI. All stimulus isolation units used were calibrated prior to each experiment and output offsets zeroed by visualization on an oscilloscope. The complete cVN electrophysiological setups used in this study are shown in Fig. 1.

### Blood collection

Approximately 5 ml of blood was collected from the left ventricle of the heart at the end of each experiment. Blood was allowed to clot at room temperature for 15 minutes prior to centrifugation at 2000 g for 20 minutes.

### LPS-induced endotoxemic shock

LPS (L2630, Sigma Aldrich) was freshly prepared the morning of each experiment by dissolving in sterile, deionized water followed by a 15 minute sonication at 37 °C. Animals were injected intravenously via a 24 G catheter inserted in the tail vein with a dose of 15 mg/kg in a total volume of 1 ml.

### Experimental Protocols

#### LPS-induced endotoxemic shock

All experiments followed a standard protocol for induction of endotoxemic shock and delivery of paired efferent stimulation and block (Fig. 1C). Animals first received 10 minutes of stimulation or paired stimulation and block (pre-stim). Upon completion, animals received either LPS or saline tail vein injections, and another 10 minutes of stimulation or paired stimulation and block were delivered (post-stim). KES block was continued through the duration of the experiment. Blood collection took place 50 minutes after the completion of the post-stim period. Recordings of cVN activity were made during the entire experiment to validate KES block of afferent activity.

#### Nerve transection studies

The cVN was transected (cVNx) in a subset of studies to characterize the effects of afferent (n = 2, data not shown) and efferent (n = 6) cVNS on the systemic response to endotoxemic shock. A cuff electrode (described above) was placed around the cVN for stimulation prior to transection. Once the cuff was secured in place, the cranial or caudal end of the nerve were cut. The nerve was stimulated pre- and post-LPS injection, and blood was collected 50 minutes after the end of the post-stim period. In addition, GSN activity was measured while stimulating the cranial or caudal ends of the transected cVN.

#### Nerve block experiments

We previously reported KES (sinusoidal) nerve block inhibited evoked potentials in the cVN, and characterized the response of the cVN to KES as a function of both KES frequency and amplitude. Based upon these findings, we used a KES frequency of 40 kHz with amplitudes in the range of 1.5–2.0 mApeak. A calibration trial was conducted to determine the specific KES amplitude for use in each experiment. The nerve was stimulated at a rate of 1 Hz and online ENG measurements were used as a readout to assess the status of KES nerve block. KES amplitudes started at 1.5 mApeak and were increased in steps of 0.1 mA_peak_ until the block threshold was identified. Both stimulation and block were turned off after identification of the block threshold. Calibration procedures lasted approximately 30–45 seconds in each experiment. Post-experiment visualization and electrophysiological assessment of nerve viability were conducted by delivering 5–10 stimulating pulses and observing evoked CAPs, along with monitoring for nerve or electrode discoloration. From all experiments conducted, nerve discoloration, but not loss of nerve conduction, was observed in 2 animals with incomplete KES nerve block and have been removed from the data pool.

### Data Analysis

#### ENG Analysis

We used ENG measurements to quantify cVNS activation and to validate the status of afferent block. All data processing and analysis was conducted in MATLAB (R2015b, MathWorks, Inc. Natick, MA). ENG recordings from the cVN and GSN were digitally band-pass filtered (100 to 3000 Hz) prior to being stimulus-triggered to generate average waveforms (20 runs per trial), resulting in a total of 210 trials per experiment. All waveforms shown in this report are averages of 20 runs unless stated otherwise. In the current experimental setup, only A and C components from stimulus-triggered average waveforms were distinguishable due to limitations in electrode-to-electrode distance. Time windows were computed using the electrode-to-electrode distance measured in the experimental setup and component-specific conduction velocities (A > 2.0 m/s, C < 2.0 m/s) for quantification of evoked components. Windows were calculated for the A and C fiber components, along with a 10 ms pre-stimulus noise window. Window bounds were set to exclude stimulus and amplifier artifacts. The root mean square (RMS) value of each window was computed using the MATLAB signal processing toolbox (rms function). The signal-to-noise ratio, represented by *θ*, for each CAP component was calculated by taking the RMS value for a given component (A or C) and dividing by the RMS value of the noise window^24^,^25^. The use of a windowed RMS metric, as opposed to peak analysis, incorporates the temporal dynamics of different CAP components and provides a more complete view of nerve activation or block. For example, small, slow-conducting fibers (e.g., C-fibers) appear as temporally dispersed waveforms which would not be captured by time of occurrence and magnitude of peaks alone.

#### Biochemical Analysis

Serum TNF-*α* concentrations were quantified using commercially available ELISA kits (BD Biosciences). Calibration curves were generated and TNF-*α* concentrations were obtained by measuring absorbance at 450 nm.

#### Statistical Analysis

Analysis of variance and t-tests were performed using the MATLAB statistics toolbox (anova1, ttest2 functions). The Jarque-Bera tests (jbtest function) was used to evaluate normality of experimental groups. All statistical tests were carried out with *α* = 0.05. All box plots show the 95% confidence interval (pink) for the mean (center bar) and 1 standard deviation (blue).

## Additional Information

**How to cite this article**: Patel, Y. A. *et al*. Kilohertz frequency nerve block enhances anti-inflammatory effects of vagus nerve stimulation. *Sci. Rep.* **7**, 39810; doi: 10.1038/srep39810 (2017).

**Publisher's note:** Springer Nature remains neutral with regard to jurisdictional claims in published maps and institutional affiliations.
