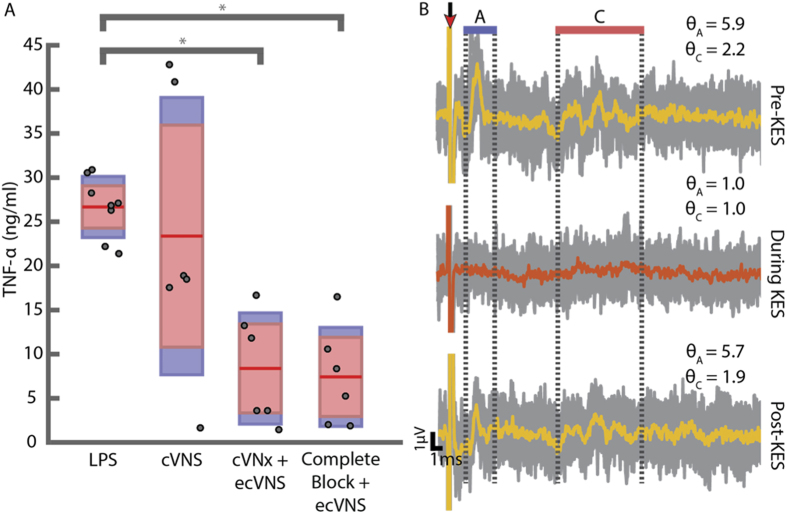# Corrigendum: Kilohertz frequency nerve block enhances anti-inflammatory effects of vagus nerve stimulation

**DOI:** 10.1038/srep46848

**Published:** 2017-06-07

**Authors:** Yogi A. Patel, Tarun Saxena, Ravi V. Bellamkonda, Robert J. Butera

Scientific Reports 7: Article number: 3981010.1038/srep39810; published online: 01 05 2017; updated: 06 07 2017

This Article contains an error in Figure 3B where the y-axis label of the scale bar is missing. The correct Figure 3 appears below as Figure 1.

## Figures and Tables

**Figure 1:**